# Sociocultural influences on asthma self‐management in a multicultural society: A qualitative study amongst Malaysian adults

**DOI:** 10.1111/hex.13352

**Published:** 2021-08-27

**Authors:** Wen Ming Koh, Ahmad Ihsan Abu Bakar, Norita Hussein, Hilary Pinnock, Su May Liew, Nik Sherina Hanafi, Yong Kek Pang, Bee Kiau Ho, Salbiah Mohamed Isa, Aziz Sheikh, Ee Ming Khoo

**Affiliations:** ^1^ Department of Primary Care Medicine, Faculty of Medicine University of Malaya Kuala Lumpur Malaysia; ^2^ Rawang Health Clinic Ministry of Health of Malaysia Malaysia; ^3^ NIHR Global Health Research Unit on Respiratory Health (RESPIRE), Usher Institute University of Edinburgh Edinburgh UK; ^4^ Department of Medicine, Faculty of Medicine University of Malaya Kuala Lumpur Malaysia; ^5^ Bandar Botanic Health Clinic, Ministry of Health Malaysia Malaysia

**Keywords:** adult, asthma, experiential learning, qualitative, self‐management, sociocultural influences

## Abstract

**Background:**

Supported self‐management improves asthma outcomes, but implementation requires adaptation to the local context. Barriers reported in Western cultures may not resonate in other cultural contexts. We explored the views, experiences and beliefs that influenced self‐management among adults with asthma in multicultural Malaysia.

**Methods:**

Adults with asthma were purposively recruited from an urban primary healthcare clinic for in‐depth interviews. Audio‐recordings were transcribed verbatim and analysed thematically.

**Results:**

We interviewed 24 adults. Four themes emerged: (1) Participants believed in the ‘hot and cold’ concept of illness either as an inherent hot/cold body constitution or the ambient temperature. Hence, participants tried to ‘neutralize’ body constitution or to ‘warm up’ the cold temperature that was believed to trigger acute attacks. (2) Participants managed asthma based on past experiences and personal health beliefs as they lacked formal information about asthma and its treatment. (3) Poor communication and variable advice from healthcare practitioners on how to manage their asthma contributed to poor self‐management skills. (4) Embarrassment about using inhalers in public and advice from family and friends resulted in a focus on nonpharmacological approaches to asthma self‐management practice.

**Conclusions:**

Asthma self‐management practices were learnt experientially and were strongly influenced by sociocultural beliefs and advice from family and friends. Effective self‐management needs to be tailored to cultural norms, personalized to the individuals' preferences and clinical needs, adapted to their level of health literacy and underpinned by patient–practitioner partnerships.

**Patient and Public Contributions:**

Patients contributed to data. Members of the public were involved in the discussion of the results.

## 1 INTRODUCTION

Globally, asthma is a common noncommunicable disease, affecting an estimated 339 million people.^1^ This number is expected to increase to 400 million by 2025,^2^ posing a significant and growing healthcare burden.^1^, ^3^ In Malaysia, asthma is among the top 20 causes of disability‐adjusted life years.^4^

Asthma self‐management refers to individuals with asthma making therapeutic, behavioural and environmental adjustments in accordance with advice from healthcare professionals.^5^ It includes discussion about self‐management and provision of a personalized written asthma action plan,^6^, ^7^, ^8^ and when supported by healthcare professionals, improves asthma outcomes such as reduced unscheduled clinic visits and use of healthcare resources, and improved asthma control and quality of life across all levels of asthma severity.^8^, ^9^ The Global Initiative for Asthma (GINA)^10^ and the Malaysian Clinical Practice Guidelines for asthma^11^ recommend that all patients should be provided with education and skills to self‐manage their asthma effectively.

Despite this, relatively few people have asthma action plans. For example, the UK National Review of Asthma Deaths showed that 33% of patients who died had no record that they sought medical assistance during their final attack; of these, only 17% had been provided with an asthma action plan.^12^ In Malaysia, only 29% of patients had an asthma action plan and of these, only half were confident about using it.^13^ A metasynthesis of qualitative data showed that medically focused action plans that did not engage the patients and include their views and preferences would remain underused.^14^

Sociocultural beliefs have been shown to influence asthma self‐management. For example, two qualitative studies from the United Kingdom highlighted the importance of social‐cultural beliefs in determining self‐management behaviour amongst minority South Asians.^15^, ^16^ Patients have their own unique beliefs and experiences, and there is a need to understand this socio‐cultural context to tailor education and support for asthma self‐management.^17^, ^18^ This has particular challenges in a multicultural country like Malaysia, in which the population is made up of three main ethnic groups, the Malay, Chinese and Indian, and native minorities, each with their own language and culture.^19^ A recent study revealed the extent to which socio‐cultural practices and traditional medicines were used to complement (or at times replaced) evidence‐based asthma management amongst Malaysian children with asthma.^13^ Building on this evidence, we aimed to explore the views, beliefs and experiences that influenced the self‐management practices of adults with asthma in the three major ethnic groups in Malaysia.

## 2 METHODS

This qualitative study was undertaken from 2018 to 2019. Ethical approval was obtained from the National Medical Research and Ethics Committee (NMRR‐18‐1087‐40634). Participants were given a participant information sheet and an opportunity to ask questions and time to consider before providing written informed consent.

### 2.1 Study setting

The study was conducted in a primary health clinic located in Klang District, Selangor, Malaysia, which is densely populated with multiracial communities and groups with various levels of income. This clinic was chosen as it runs a dedicated asthma clinic and provides emergency asthma care. About 240 people with asthma are reviewed regularly by doctors in charge of the asthma clinic; during these reviews, their asthma control is assessed, peak flows are recorded and asthma education is provided.

### 2.2 Participant recruitment

Adults (18 years and over) with physician‐diagnosed asthma were approached by the doctors in charge either during their asthma review, an emergency visit for an acute exacerbation or if they had missed a scheduled appointment at the clinic. We used purposive sampling to achieve a maximum variation sample based on age, gender, ethnicity, asthma duration and ownership (or not) of an asthma action plan. Participants who agreed to participate were given a date and time for an interview that was convenient to them. They were offered an honorarium of RM 100 (≈ £18.50) to cover their time and travel expenses.

### 2.3 Data collection

To provide context and characterize the participants, we collected basic sociodemographic, asthma duration and control based on GINA symptom control^10^ using a self‐completed questionnaire.

We used a topic guide to facilitate individual in‐depth interviews that aimed to understand participants' personal illness experiences. The topic guide was developed based on the Theory of Planned Behaviour,^20^ the known features of asthma self‐management, including how they learnt to live with a variable illness, attitudes to taking preventer medication, how they recognize and respond to worsening asthma symptoms, signs (and peak flows) and the use (if any) of an asthma action plan.^21^, ^22^ The Theory of Planned Behaviour has been widely used in predicting and explaining self‐management behaviour.^23^ The topic guide is provided in the Supporting Information S1.

The interviews lasted up to 90 min and were audio‐recorded. The researcher (W. M. K.) conducted the interviews in the participants' preferred language, Malay, English or Mandarin or a mixture of these languages (as is the norm in Malaysia). During the interview, W. M. K. encouraged participants to expand and elaborate on their views to gain a better understanding of the responses given. Inhalers, peak flow meters and spacers/aero‐chambers and asthma action plans were shown to the participants to avoid any misunderstandings. Field notes were made to capture nonverbal cues, gestures and emotions of the participants, and to record devices and medications that participants referred to. This information was incorporated into the verbatim transcripts to add context to the participants' views. Interviews were continued until data saturation was reached with respect to views on living with asthma.

### 2.4 Data analysis

Data were managed and coded using NVivo version 11.4.3. An interpretative–descriptive approach to thematic analysis was used to describe and understand the views and experiences of participants on asthma self‐management.^24^ The first two transcripts were coded independently by three authors (W. M. K., E. M. K., A. I. A. B.) and an agreed initial list of codes was developed. Next, W. M. K. used this coding framework to code all the remaining transcripts. Any new codes that emerged were added into the existing coding framework and checked by E. M. K. and A. I. A. B. Any discrepancies were resolved by consensus to produce a final coding frame. All codes were then categorized into themes.

Interviews were transcribed by a transcriber fluent in the appropriate language, coded and analysed by the trilingual researcher (W. M. K.) in the language(s) in which the interview was conducted. Malay‐ and Mandarin‐language transcripts were translated into English by an independent translator. Representative quotes related to the research questions were selected for the paper.^25^

### 2.5 Reflexivity

W. M. K. is a primary care physician. Throughout 8 years of work experience, she has managed many cases of asthma with varying degrees of control. The researcher postulated that many of the patients had poor knowledge about asthma self‐management and poor adherence to asthma medications, which might have led to her making assumptions about the participants while conducting the study and analysing the data. The use of a field journal that documented the researcher's reflections, thoughts and ideas generated when in contact with the participants reminded the researcher to be aware of these assumptions and constantly reflected on their influence on the study.

### 2.6 Saturation and transferability considerations

Data saturation was reached by the 21st interview, by which no new information or themes were observed in the data. However, interviews were carried on till the 24th interview to further confirm data saturation. Participants recruited were of three major ethnic groups (Malay, Chinese and Indians) that made up the major populations in Malaysia; their social and cultural practices were assimilated to a large extent.

## 3 RESULTS

Twenty‐four participants were interviewed. Table 1 summarizes their characteristics.

**Table 1:** Characteristics of the participants

Characteristics	Number (*n* = 24)
Age (years)	
18–30	5
30–40	6
40–50	5
50–60	3
60–70	5
Gender	
Male	10
Female	14
Ethnicity	
Malay	10
Chinese	7
Indian	7
Education level	
No formal education	1
Primary	3
Secondary	14
Tertiary	6
Occupation (ISCO‐08)^26^	
Professionals	5
Clerical support worker	1
Services and sales worker	2
Craft and related trade worker	4
Plant and machine operators	3
Others (housewife, retiree)	9
Ownership of asthma action plan	
Yes	6
No	18
Asthma duration (years)	
1–5	4
5–10	7
10–20	4
>20	9
GINA asthma control^a^,^10^	
Well controlled	2
Partly controlled	11
Uncontrolled	11

Abbreviations: GINA, Global Initiative for Asthma; ISCO‐08, International Standard Classifications of Occupations.^26^

^a^ GINA^10^ classifies asthma control according to responses to four questions (in the past 4 weeks, has the patient had: Daytime asthma symptoms more than twice/week; any night waking due to asthma; reliever needed for symptoms more than twice/week; and any activity limitation due to asthma?). Well controlled is defined as ‘None of these symptoms’; Partly controlled is defined as ‘1–2 symptoms’; and uncontrolled is defined as ‘3–4 symptoms’ in the past 4 weeks.

Four themes emerged: (1) the ‘hot and cold’ concept of asthma self‐management; (2) poor understanding of asthma and misconception on asthma control and its treatment; (3) variable support on asthma self‐management by healthcare practitioners; and (4) social influences on asthma self‐management.

### 3.1 The ‘hot and cold’ concept of asthma self‐management

Participants viewed this concept in two ways. One was the belief in an inherent body constitution of ‘hot or cold’ that contributed to asthma, while the other was the ambient temperature of ‘hot’ or ‘cold’.

From this perspective, one aim of self‐management was to neutralize the ‘hot or cold’ body constitution by eating food that was believed to have opposite properties. Complementary medication was often based on the belief of inherent ‘hot and cold’ properties of food that neutralized the ‘cold’ effect of asthma.

*If ‘heaty asthma*’*, you take that ginger with that liver then you will become worse. So, before* (previously)*… soya beans, kangkong, tofu, that salted vegetable I cannot take… Now I build up my body… is heaty already… So, cooling… not much effect ah… now soya beans, just everything I can take…*. (60–70 years old/Chinese/retiree/uncontrolled asthma)

*The villagers* (told me)*, because these faeces are ‘hot*’*, and because goat is ‘hot*’*, my body is cold, eating (goat) faeces will warm up my body… but it didn't work …*. (40–50 years old/Malay/driver/uncontrolled asthma)

Some viewed the ‘hot and cold’ concept as differences in temperature such as hot or cold food and beverages, as well as washing, which caused coldness in the hands. If asthma symptoms were considered to be due to cold, strategies were used to ‘warm up’ the body with hot food, hot rubs, use of gloves and socks and adjusting the timing of showers to avoid cold.

Food and beverages that are cold, such as ice‐cream and cold drinks, were identified as asthma triggers from past experience and were avoided to prevent attacks.

*When I get the wheezing and difficulty of breathing… canned milk, one spoon, put hot water to make up to one cup, 500mls to 200mls, mix it and drink. Reduces* (symptoms) *a bit*. (18–30 years old/Indian/housewife/uncontrolled asthma)

### 3.2 Lack of understanding of asthma and misconceptions about asthma control and its treatment

There was often a lack of understanding about asthma and misconceptions about the treatment based on their beliefs, family advice and previous experience. There was concern about the side effects of inhaler use that included headache, kidney damage, carcinogenicity and fear of dependency.

*The blue colour pump* (salbutamol) *is to open my lungs and after 15 minutes you must use the red colour pump* (budesonide) *to close. If* (my lungs are left) *open, I will vomit*. (30–40 years old/Indian/factory worker/partly controlled asthma)

*… I don't want to be too dependent on this medication* (salbutamol inhaler)*… if keep depending on it, I will always get* (attacks)*…*. (18–30 years old/Malay/housewife/partly controlled asthma)

*… I stopped budesonide… because my relative tells me it's going to give me cancer*. (40–50 years old/Chinese/teacher/uncontrolled asthma)

There were misconceptions about what good control could be and some described adapting to and living with symptoms and were unaware that they could prevent or relieve them.

*We feel sometimes when there is wheeze sound, it is normal. No need to use inhaler also OK*. (40–50 years old/Malay/Tailor/partly controlled asthma)

Only six of the participants were familiar with asthma action plans. Most, even when shown an action plan, had very limited understanding of how it could help them manage their asthma. Some participants regarded asthma action plans as a ‘meal plan’ with advice on foods to avoid because they triggered asthma symptoms. Others could not recall the details in the asthma action plan and the actions that they should take when experiencing changes in their asthma symptoms. Several people identified barriers to action plan use in terms of both the content and practicalities.

*From what I understand, this plan is for asthma prevention lah (a suffix used to emphasise the previous words) …*. (40–50 years old/Malay/teacher/well‐controlled asthma)

*The red area* (information in a traffic‐light‐coded action plan) *was good. But doesn't tell you that should you use or continue using your normal medication or not*. (40–50 years old/Chinese/teacher/uncontrolled asthma)

*No, I am illiterate, how to read?* (60–70 years old/Chinese/housewife/uncontrolled asthma)

### 3.3 Variable support for self‐management by healthcare practitioners

Some healthcare practitioners considered the beliefs and views of patients with asthma while providing asthma education and advice on asthma self‐management, but more often, participants reported variable advice. Examples included a 31‐year‐old, Indian, factory worker who was advised to stop jogging and dancing because she had asthma, and a 41‐year‐old housewife who was told to ensure that she avoided getting wet when doing chores to prevent attacks.

Poor communication and ineffective doctor–patient partnerships impeded knowledge transfer and sometimes resulted in people seeking alternative care.

*Because we can't express* (our thoughts)… (and the doctor said) *‘Are you the doctor, or I am the doctor?*’*… Sometimes we want to tell but they straight away stop us… Why did we want to go there* (traditional practitioner) *to get asthma medications? Because doctors here, not all but some of them, do not communicate with patients*. (30–40 years old/Indian/housewife/uncontrolled asthma)

*For me, everything I did is wrong, if the doctor really advised me the ways for daily living … from now on lah… maybe I will follow lah, because I also want to follow lah…*. (30–40 years old/Chinese/police/partly controlled asthma)

*I asked for the doctor's advice…'can I buy the nebulizer machine?' Doctor told me… ‘We need to monitor you', ‘you cannot use it yourself every time when you have an asthma attack, only neb neb neb… cannot lah…*. (40–50 years old/Indian/housewife/uncontrolled asthma)

### 3.4 Social influences on asthma self‐management

Family and friends influenced participants' self‐management of asthma, in particular, the nonpharmacological approach, the usage of complementary and alternative medications and over‐the‐counter purchase of medications from community pharmacies.

*She* (my neighbour) *came to my house. She asked why (are you wheezing) sister?*

*Go and take this medication (prednisolone, that can be purchased over the counter from the community pharmacy), try and see… she* (my neighbour) *says…*. (40–50 years old/Malay/housewife/uncontrolled asthma)

*My children ask me to walk…my neighbour also walked… I walk lah… if diabetes is able to disappear, asthma can also disappear …*. (60–70 years old/Malay/retiree/partly controlled asthma)

There was perceived social stigma with the use of inhalers as a self‐management strategy. One participant felt embarrassed to use an inhaler in public and resorted to smoking for symptom relief during an exacerbation.

*Because my job involves the public right, so if I want to use it* (the inhaler)*, I don't use in front of the public as they will see, hide yourself, in the car or something*.

*If I have difficulty breathing, I will use this method that makes the mouth small* (pursed lip breathing) *… If it doesn't go away, I smoke one cigarette, I will tell my subordinate, one cigarette…smoke only*. (30–40 years old/Chinese/police/partly controlled asthma)

## 4 DISCUSSION

### 4.1 Principal findings

Our participants had learnt over time to self‐manage their asthma in the context of the healthcare norms of Malaysian society. The ‘hot and cold’ concept is widely accepted by all the cultural groups in Malaysia and many of the misconceptions about asthma were based on this. Medications were used experimentally, and self‐management was based on personal beliefs, perceptions, past experience and information obtained from family, friends and healthcare practitioners. This resulted in patients developing their own self‐management plan rather than following the plans recommended by asthma guidelines. Furthermore, stigma attached to inhaler use further reduced compliance and use of action plans.

### 4.2 Strengths and limitations

A strength of our study is that we explored understanding of asthma self‐management in a multiethnic and culturally diverse setting by conducting interviews in three languages, followed by analysis to ensure that we did not miss cultural nuances. Although our participants came from a range of socioeconomic backgrounds, one limitation is that we only recruited participants from the public health clinic and our findings do not reflect the views of patients from the private healthcare sector. We reached data saturation with respect to the opinions of the three major ethnic groups, but did not recruit natives, foreigners or migrant workers living in Malaysia, so we cannot comment on their cultural perspectives and whether they conform to the cultural norms that we observed.

This study focused on the perspectives of people living with asthma, but exploration of asthma self‐management from the healthcare provider point of view could further enhance understanding of asthma self‐management among patients with asthma. The researcher who undertook the interviews and analysed the data is a primary care physician, but we were aware of reflexivity and the emerging findings were discussed within a multidisciplinary team to ensure a balanced interpretation.

### 4.3 Interpretation of findings

The ‘hot and cold’ concept of health and illness appears in many geographically separated areas of the world. The origins of this concept are debated, with European accounts tracing the origin to Hippocrates and Galen, which influenced the physicians of medieval Islam and later spread to America. Similar traditions, however, were seen in early Chinese and Indian cultures.^27^ Although varying in detail, the common concept is that health can be lost or restored as a result of the effect of ‘hot’ and ‘cold’ elements on the human body.^28^ These elements do not refer to the actual temperature or taste but rather the internal properties of the foods or herbs as well as the state of the illness. Based on this concept, health practices were targeted at the ‘avoidance of extreme conditions’ or consumption of food items to restore the balance between the opposing forces of ‘hot and cold’. In the Asian context, the ‘hot and cold’ health belief stemmed from traditional ancient Chinese medicine that viewed Yin and Yang as the symbolic power of hot and cold that appeared to be concurrently dependent on and in opposition to each other.^29^ A person is said to have perfect health if he or she has an unopposed flowing chi with well‐balanced Yin–Yang forces. This balance can be restored by dietary changes or the use of traditional medications and herbs to compensate the effect of excess ‘hot’ or ‘cold’ elements. Similarly, in Indian culture, the ‘hot and cold’ concept had long been practiced by the people in Sri Lanka, using food to achieve balance between the ‘hot’ and ‘ cold’ elements based on Ayurvedic medicine in disease prevention and treatment.^30^

The ‘hot and cold’ health belief is an accepted cultural norm in Malaysia, and is practised across all major ethnic groups (Malay, Chinese, Indian) as a result of cultural assimilation.^31^ A qualitative study conducted among carers and children with asthma in Malaysia showed that some carers used certain ‘hot’ complementary and alternative medicines to counteract asthma, which is believed to be ‘cold’.^16^ Healthcare practitioners need to acknowledge these cultural beliefs so that asthma self‐management can be discussed in terms that patients can understand. Whilst of obvious importance in an explicitly multiethnic country (such as Malaysia), globalization means that cultural diversity is now the norm in almost all societies and an understanding of a widely held belief (such as ‘hot/cold’ concepts) is of relevance universally.

Culturally targeted or tailored asthma self‐management interventions could potentially enhance acceptance of the intervention, reducing unscheduled visits and improving asthma control and confidence in self‐managing asthma.^18^ On a practical level, tailoring asthma education materials by appropriate use of ‘hot and cold’ colours and colour‐coded inhalers with ‘hot’ colour tones would resonate with these widely held ‘hot and cold’ cultural beliefs.^32^

Few of our participants had an action plan, suggesting underprovision by the healthcare practitioners. One reason may be concerns about poor health literacy; indeed, many older Malaysians (including one of our participants) are illiterate.^33^ To assist patients with low health literacy levels, a pictorial action plan for patients may need to be considered.^34^ In addition, healthcare practitioners, nurses and pharmacists need to be trained to educate people with asthma irrespective of their level of literacy.^35^, ^36^

Key barriers to provision of supported asthma self‐management are poor communication between the doctor and the patient and the lack of continuity of care, which frustrated and confused our participants. Similar findings were also noted in a systematic review and thematic synthesis focusing on barriers and facilitators of effective self‐management in asthma.^37^ These barriers might further be due to the lack of time, organizational barriers and the need for further training in professional skills development.^38^ Good doctor–patient partnership, communication and continuity of care are central to effective supported asthma self‐management.^10^, ^29^ This could be enhanced by patient‐centred consultation skills and shared decision‐making, providing opportunities for patients to voice their expectations and concerns and to cocreate their own asthma management plan.^7^, ^39^

Stigma led to at least one of our participants preferring to smoke rather than using an inhaler during an attack. This has been described before and attributed to the fact that smoking is accepted or seen as a social norm in Malaysia and Thailand and widely accepted among young adult males.^40^ In Australia, studies have shown that close to half of adult patients felt embarrassed to use inhalers in public as it revealed the diagnosis of asthma.^41^ This stigma hinders adherence to long‐term management as well as everyday life and ability to socialize,^42^ and is associated with increased morbidity.^43^ Whilst patients could be imparted skills to overcome the stigma they face, it is important to educate the public and community about asthma and its treatment to reduce the stigma of inhaler use among the public and patients. A whole‐system approach is required to achieve sustained improvements in clinical asthma outcomes.^44^

### 4.4 Implications for clinical practice

Supported self‐management in asthma has gained recognition over the years based on extensive evidence of its effectiveness in reducing asthma morbidity and mortality.^6^, ^7^, ^8^, ^9^ Supported asthma self‐management includes a written asthma action plan, self‐monitoring and regular review by a knowledgeable healthcare provider.^10^ Our findings highlight some of the challenges faced by primary care providers in a multicultural country such as Malaysia. Personalization of an asthma action plan involves not only incorporation of individual patients' preferences but also tailoring to the embedded cultural practices and concerns. This can be achieved by better collaboration between healthcare practitioners and patients, and developing the skills of shared decision‐making, acknowledging cultural norms whilst empowering patients with the confidence, skills and knowledge that they need to adopt evidence‐based self‐management on a regular basis. Accurate and easy to understand information on asthma, potentially delivered through pictorial representation, should be made widely available in clinics and pharmacies, and community awareness programmes may be helpful. Patient peer support groups, family and friends may reach out to communities where health literacy is low.

## 5 CONCLUSIONS

Asthma self‐management practices were learnt experientially from personal experiences and beliefs that are culturally ingrained among patients with asthma in Malaysia, influenced by healthcare practitioners, family and friends. Effective self‐management needs to be tailored to the communities' cultural norms (especially the ‘hot and cold’ beliefs), personalized to the individuals' preferences and clinical needs, adapted to their level of health literacy and underpinned by robust patient–practitioner partnerships.

## CONFLICT OF INTERESTS

 E. M. K. reports grants from the National Institute for Health Research (NIHR) Global Health Research Unit on Respiratory Health (RESPIRE) and Seqirus UK; personal fees from AstraZeneca and GlaxoSmithKline; and is board director of the International Primary Care Respiratory Group.

A.S. is the primary investigator on RESPIRE grant from NIHR.

All other authors declare that there are no conflict of interests.

## AUTHOR CONTRIBUTIONS

Aziz Sheikh was PI of RESPIRE contributing to the conception, design and oversight of this study and contributed to interpretation and editing of the study manuscript. All authors contributed to the conception of this study; W. M. K., E. M. K. and A. I. A. B. performed the data collection, coding and data analysis. W. M. K. drafted the manuscript, and all authors provided recommendations to the editing and revision of the manuscript. All authors approved the final version.

## ETHICS APPROVAL

Medical Research and Ethics Committee (MREC), Ministry of Health Malaysia (MOH): NMRR‐18‐1087‐40634 and sponsorship approval from the Academic and Clinical Central Office for Research & Development (ACCORD) at the University of Edinburgh (AC19040).

## Supporting information

Supporting information.

## Data Availability

Data of relevant findings are shared as deidentified quotes in the paper. Other data are available on request from the authors.
