# Supplementary material for: Sociocultural influences on asthma self‐management in a multicultural society: A qualitative study amongst Malaysian adults
Source: Health Expect. 2021 Aug 27;24(6):2078–86. doi: 10.1111/hex.13352 (PMC8628588; doi:10.1111/hex.13352)
Supplement: Supplementary file 1 — Supporting information. [file HEX-24-2078-s001.docx]

**Online Supplement 1:**

Interview Topic Guide

**Preamble:**

- Ice-breaking and explain aim of the study
- Explain that there is no right or wrong answer
- Explain need to get consent for the interview and audio-recording

| **Questions** | **Prompts** |
| --- | --- |
| **Views and Experiences:**  **Asthma Control, on Normal Days and Exacerbations** | **How is your asthma normally? (to elicit good control, bad control, attack)** Is that normal for you? If yes, why? If no why?  **What do you think of your asthma control?** If good, why? If not, why?  **What do you normally do if your asthma is good?**  **What do you normally do if your asthma is bad?**  Have you ever had an asthma attack? What is it? What do you do when you have an attack?  **When or on what circumstances do you decide to seek medical attention?**  **What do you do on normal days to prevent an asthma attack?**  What do you think on the way of you managing asthma?  What do you think should be done?  Any other problems you encounter when you manage your asthma? What do you do if you have problems in managing asthma?  What are the things you think that will make it more difficult or prevent you in managing asthma? |
| **Views and understanding of Asthma Action Plan** | **Have you heard of asthma action plan?** (If yes), can you tell me more about this plan? How do you use it in managing your asthma? How can it help you in managing your asthma?  (If no), do you use any guide to manage your asthma? If yes, what is that? From where have you heard it? How does it help you? if not, how do you manage your asthma?  **Are there anyone around to support you to manage your asthma?**  (If yes), who are they? How do they support you in managing your asthma? Do they influence you manage your asthma? If Yes? how? If No, why not?  (If no), why? Would you like to have support to manage your asthma? If so, on what aspect? How will that help you?  **What do you need to help you in managing asthma?** |
| **Medications (inhalers)** | **What do you use for asthma? (show inhalers)**  (If inhaler), which one? And what is the purpose of each one?  **How do you use it? Do you feel it works?**  **When do you use the inhaler(s)?**  **When you have an attack, what do you do?** |
| **Spacer** | **Do you have this? (shows spacer or aero-chamber)**  **What is the purpose of this device?**  **When do you use it?**  **How do you find it helping you in self-managing your asthma?** |
| **Cultural practice and complementary medicine** | **Is there anything else you do to control your asthma?**  **Besides medications prescribed by your doctors, do you take anything else to help your asthma?** |
| **Views on self-management** | **What do you think about the way you self-manage your asthma?**  **Are there any areas you wanted to improve? Why?**  **Do you think it is important to self-manage your asthma? If yes, Why? If no, Why not?**  **What are the ways to help in self-managing asthma?** |
| **Wrap Up** | **Is there anything you would like to add before we end this session?** |
